# Do palliative care patients and relatives think it would be acceptable to use Bispectral index (BIS) technology to monitor palliative care patients’ levels of consciousness? A qualitative exploration with interviews and focus groups for the I-CAN-CARE research programme

**DOI:** 10.1186/s12904-022-00949-w

**Published:** 2022-05-24

**Authors:** Anna-Maria Krooupa, Patrick Stone, Stephen McKeever, Kathy Seddon, Sarah Davis, Elizabeth L. Sampson, Adrian Tookman, Jonathan Martin, Vinnie Nambisan, Bella Vivat

**Affiliations:** 1 Division of Psychiatry, Marie Curie Palliative Care Research Department, University College London, London, UK; 2 Department of Nursing, The University of Melbourne, Melbourne, Australia; 3 Marie Curie Palliative Care Research Voices, London, UK; 4 Division of Psychiatry, University College London, London, UK; 5 Marie Curie Hospice Hampstead, London, UK; 6 Field Editor Cochrane; Palliative and Supportive Care, Oxford, UK; 7 Central & North West London NHS Foundation Trust, London, UK; 8 National Hospital for Neurology & Neurosurgery, University College London Hospitals NHS Foundation Trust, London, UK; 9 HCA (UK), London, UK

**Keywords:** Palliative care, Terminal care, Hospices, Consciousness monitors, Hypnotics and sedatives, Qualitative research, Focus groups

## Abstract

**Background:**

Bispectral index (BIS) monitoring uses electroencephalographic data as an indicator of patients’ consciousness level. This technology might be a useful adjunct to clinical observation when titrating sedative medications for palliative care patients. However, the use of BIS in palliative care generally, and in the UK in particular, is under-researched. A key area is this technology’s acceptability for palliative care service users. Ahead of trialling BIS in practice, and in order to ascertain whether such a trial would be reasonable, we conducted a study to explore UK palliative care patients’ and relatives’ perceptions of the technology, including whether they thought its use in palliative care practice would be acceptable.

**Methods:**

A qualitative exploration was undertaken. Participants were recruited through a UK hospice. Focus groups and semi-structured interviews were conducted with separate groups of palliative care patients, relatives of current patients, and bereaved relatives. We explored their views on acceptability of using BIS with palliative care patients, and analysed their responses following the five key stages of the Framework method.

**Results:**

We recruited 25 participants. There were ten current hospice patients in three focus groups, four relatives of current patients in one focus group and one individual interview, and eleven bereaved relatives in three focus groups and two individual interviews. Our study participants considered BIS acceptable for monitoring palliative care patients’ consciousness levels, and that it might be of use in end-of-life care, provided that it was additional to (rather than a replacement of) usual care, and patients and/or family members were involved in decisions about its use. Participants also noted that BIS, while possibly obtrusive, is not invasive, with some seeing it as equivalent to wearable technological devices such as activity watches.

**Conclusions:**

Participants considered BIS technology might be of benefit to palliative care as a non-intrusive means of assisting clinical assessment and decision-making at the end of life, and concluded that it would therefore be acceptable to trial the technology with patients.

**Supplementary Information:**

The online version contains supplementary material available at 10.1186/s12904-022-00949-w.

## Background

Patient comfort and symptom control are central to palliative and end-of-life care, and the quality of that care [1–4]. Sedative medications may be helpful for managing intractable symptoms when people are dying [5, 6]. Clinical practice guidelines on the use of sedatives in palliative care generally recommend their proportionate use for distressing symptoms [7]. This entails accurate and effective monitoring of patients’ levels of consciousness [8].

Previous research has found that clinicians mostly assess levels of consciousness of sedated patients using clinical judgement and observation [9, 10], occasionally supported with structured observer rating scales [11]. However, none of the existing scales have been fully validated for use in palliative care [12]. Moreover, observational methods alone may not reliably assess patients’ levels of consciousness, because they are dependent on subjective judgements and interpretations of patients’ signs and responses. Several studies have suggested that a person’s lack of responsiveness due to reduced consciousness may not indicate that they lack awareness [13–15], while systematic differences have been found in how health care professionals and patients’ relatives estimate patients’ symptom severity [16]. Inadequate assessment may impede effective sedative use in palliative care, and result in sub-optimal care [17].

Novel technological approaches have potential for assessing and monitoring palliative care patients’ physiological status and symptoms [18, 19]. An ethnographic study of end-of-life care in highly technological environments, such as intensive care units (ICUs), proposed that a number of factors, beyond the presence of technologies alone, shape perceptions of the use of medical technologies at the end of life [20]. These include whether the expected outcomes are delivered, and whether the technologies are easy to use and understand [20]. Palliative care health professionals may be sceptical about integrating such technologies into existing services, perceiving them as potentially disruptive to current caring practices based on face-to-face communication and physical contact [21, 22]. However, one recent palliative care study found that patients and carers were open to the use of technology at the end of life [23]. Another study exploring the use of remote technologies for monitoring palliative care patients’ symptoms at home found that patients and carers reported feeling reassured by these technologies [24]. Participants in a study of palliative care patients’ and carers’ perceptions of telehealth applications commented that such initiatives should be offered as a supplement to existing practice [25].

Technological measures of physiological parameters may be helpful adjuncts to clinical assessments of levels of consciousness. Depth-of-anaesthesia technologies drawing on electroencephalogram information are already used to enable non-invasive monitoring of levels of consciousness of patients undergoing general anaesthesia [26]. To date the most widely studied such technology is Bispectral index (BIS) monitoring [27]. BIS monitors obtain electroencephalogram information by means of a sensor applied to the forehead, and this information is translated into a dimensionless number ranging from 0 to 100, using a commercial algorithm. Numbers correlate with clinical states and expected responses, with lower numbers indicating lower levels of consciousness [28]. BIS has been validated with patients receiving general anaesthetics [29] and is recommended for preventing over- or under-medication of high-risk patients under general anaesthesia [30]. Its usefulness has been explored in settings including endoscopy [31, 32], ICU [33], and emergency departments [34, 35]. However, BIS is not currently a component of usual care when monitoring or titrating sedation for palliative care patients.

No studies exploring BIS use for UK patients receiving palliative care have yet been conducted. A few studies in other countries have explored the potential for using BIS in palliative care [36–40], providing some evidence to support the utility and applicability of BIS monitoring in the palliative care context. However, these studies used the technology with unconscious patients, as did a recent study investigating the use of a different monitoring device with unconscious sedated palliative patients [41]. Six and colleagues found that no family members reported any concerns with the appearance of the sensor [41]. They concluded that the use of non-invasive monitoring technologies in palliative care should be considered for their potential to improve patient care [41].

No previous studies of BIS or of other technological devices for assessing levels of consciousness have systematically investigated the acceptability of the technology to palliative care patients and their relatives in advance of using it clinically. However, all previous studies have obtained informed consent from patients/family members prior to using the technologies [39–41]. In contrast, our series of studies of the use of sedatives and BIS technology in palliative care in the UK included an exploratory study, investigating the acceptability of the technology in advance of introducing it into the clinical context, conducted as part of a doctoral project [42]. This study, reported here, explored the acceptability of trialling BIS in clinical practice through a qualitative investigation into what current patients, relatives of current patients, and bereaved relatives thought of this technology and its potential use with palliative care patients. Alongside this, we conducted a study examining current sedation practices through interviews with clinicians and an analysis of patient records [9].

## Methods

### Aim

This study aimed to explore the views of current palliative care patients, relatives of current patients, and bereaved relatives about the acceptability of trialling BIS technology with patients in practice. Our primary aim was to establish whether patients and/or carers had any reservations which were sufficiently strong to indicate that we should not proceed to the proposed next phase of the overall study: exploring the use of BIS in practice.

### Design

We employed a qualitative design, primarily using focus groups, plus individual semi-structured interviews for those participants who were unable or unavailable to attend focus groups. We analysed data using the Framework method [43]. We were not seeking to obtain an exhaustive, theoretically generalisable insight into participants’ perceptions of the technology, not least because they were unable to see the technology being used directly, so were only able to hypothesise about its use in practice. In addition, lack of prior research in this area meant that it was impossible to identify potentially relevant characteristics for which we might sample potential participants, while representative sampling of palliative care patients is often more challenging than for other patient groups. This paper follows the standards for reporting qualitative research (SRQR) guidelines [44].

### Setting and recruitment

Potential participants were identified through a collaborating hospice, using a mixture of convenience and snowball sampling [45]. Different approaches were used for each of the three groups:

- Patients: The hospice clinical team used criteria identified by the research team (Table 1) to screen patients for eligibility. The researcher then approached all patients identified as suitable.
- Current relatives: Clinicians identified patients with relatives who might wish to participate and asked those patients to request their relative’s permission for the researcher to approach them. Previous patient participants were also asked to recommend relatives who might be willing to take part (i.e., ‘snowballing’).
- Bereaved relatives: The hospice bereavement coordinator identified people who were suitable to approach, on the basis of previous and/or recent interactions, and related perceptions of how distressing contact might be. Initial screening was of relatives who had been bereaved between 4 and 11 months previously, following the practice of the VOICES national survey of bereaved carers [46]. These individuals were sent letters of invitation, with the study information sheet enclosed. The initial response rate was low, so inclusion was later extended to relatives bereaved up to 22 months previously, and a further round of letters was sent.

**Table 1:** Eligibility criteria for study participants

**Inclusion criteria**
• Adults, over 18 years of age • People receiving palliative care OR Relatives of people currently receiving palliative care OR Relatives of people who have died under the care of a palliative care team 4–22 months previously • Able to communicate in English • Well enough to participate in a discussion for 30–60 min (as deemed by the attending clinical team)
**Exclusion criteria**
• People who cannot speak English • People with cognitive or communicative difficulties

All potential participants were invited to participate in a focus group with three or four other people, with separate groups for patients, current relatives, and bereaved relatives. Anyone who was interested but unable or unavailable to take part in any scheduled focus group was offered the option of an individual interview. Focus groups and interviews were held either at the hospice or the university.

### Data collection

Focus groups and interviews were conducted between February and December 2017. All participants provided written informed consent prior to participation. One researcher (AMK; then a doctoral student) facilitated the focus groups, another (BV; an experienced qualitative and palliative care researcher) observed. Both conducted individual interviews, depending on participant and researcher availability.

The project team developed a topic guide (Additional file 1) in line with recommendations for conducting qualitative research in the palliative care setting [47, 48]. The topic guide explored participants’ existing knowledge and/or prior experiences of medications with sedating effect and of monitoring levels of sedation/consciousness, thoughts about using BIS in palliative care, and views about an acceptable duration of BIS monitoring*.* A wider Study Advisory Group of palliative care clinicians and service user representatives reviewed, provided feedback, and approved the final version of the topic guide.

Each focus group or interview began by demonstrating the BIS technology, explaining its functioning, and outlining its limited use in palliative care, and the limits to existing knowledge (as also noted in the participant information sheet). This led to discussion of the technology, participants’ views on it, their prior experiences, if any, and their understanding of sedation practice.

We collected sociodemographic data from all study participants, and relevant clinical data from patient participants. All participants chose a pseudonym for the interviews/focus groups and data collection forms, in line with the NHS Code of Confidentiality [49]. Interviews and focus group discussions were audio-recorded on a password-protected recording device, and transcribed verbatim for analysis, after which audio files were deleted.

### Data analysis

Our study focused specifically on participants’ responses to the possibility of trialling BIS technology with current palliative care patients, and their perceptions of this technology. This was so as to provide us with information on whether it would be acceptable to proceed with exploring BIS use with current patients, the proposed next stage of the study. We analysed interview data using the Framework method, which is particularly suitable for qualitative health research [50], enabling both inductive and deductive theme development. We followed the five key stages of the Framework approach: familiarisation with the data; identification of a framework; indexing; charting; and mapping and interpretation [43]. Our framework was chiefly that of the topics in the topic guide, with scope to include additional themes, if any should be identified.

Transcripts were uploaded into QSR NVivo (version 11), and coded. Two researchers (AMK, BV) iteratively defined, compared, and contrasted initial codes and categories within the framework, with ongoing discussions on these. The Study Advisory Group provided further insight and comments. AMK derived sub-themes and themes, and mapped relationships between these and the codes and categories, assisting interpretation and conceptualisation. These were discussed further with the Study Advisory Group, and reported in AMK’s thesis [42]. For the current paper we revisited the analysis and agreed some further refinement of the themes and sub-themes.

## Results

We conducted 7 focus groups and 3 individual interviews, with a total of 25 participants: 10 patients, 4 current relatives, and 11 bereaved relatives. All our participants had personal experience of living with a terminal illness or of caring for someone in such a position, but most had no professional experience of providing health or social care, although one was a nurse. Twenty-two people participated in the seven focus groups: ten patients in three groups; three current relatives in one group; and nine bereaved relatives in three groups. The three individual interviews were with one current and two bereaved relatives. Table 2 presents participant information.

**Table 2:** Participant characteristics

Characteristics	Patients (*n* = 10)	Current patient relatives (*n* = 4)	Bereaved relatives (*n* = 11)
**Age Group**			
18–34	–	–	–
35–44	–	1	–
45–54	2	–	5
55–64	2	2	–
65–74	4	–	4
75 +	2	1	2
**Sex**			
Female	4	2	8
Male	6	2	3
**Present work situation**			
Working full-time hours	–	2	3
Working part-time hours	1	1	2
Unemployed/Job seeking	–	–	1
Medically retired	4	–	–
Retired	5	1	5
**Performance status**^a^			
0: Fully active	–	–	–
1: restricted in strenuous activity but ambulatory	1	–	–
2: Symptomatic, < 50% in bed during the day	3	–	–
3: Symptomatic, > 50% in bed, but not bedbound	4	–	–
4: Completely disabled	2	–	–
**Time since family member passed away**			
6–12 months	–	–	5
12–18 months	–	–	4
18 + months	–	–	2

^a^World Health Organization (WHO) performance status classification [51]

The median duration of the focus groups was 58 min (range: 42–82 min), and the three interviews lasted 42, 43, and 46 min. We identified three main themes from these discussions: (1) Prior knowledge and/or experience of sedation, (2) Attitudes towards BIS, and (3) Acceptable durations and settings for BIS use.

### Theme 1: prior knowledge and/or experience of sedation

This theme comprised one principal sub-theme: “experience of sedative medications”, and one minor sub-theme: “sedation monitoring”. Τhe principal sub-theme arose with almost all participants, who, in various clinical settings, including palliative care, had either personally received medication with sedative effects for anxiolysis, analgesia or procedural sedation, or observed friends or relatives who had. These participants’ perceptions about medicines with a sedating effect were mixed. Some reported having benefited from the use of sedatives, and described experiencing reduced anxiety and fear after taking such medications:

I’ve had sedation and it was perfectly pleasant. I was extremely relaxed, and it took any kind of fear away. (Ellie, bereaved relative, 65-74)

Others had negative views, mainly arising from feeling that sedative medications had not adequately controlled their symptoms:

I didn’t really find the sedatives sedating, they didn’t make me sleep at all, kept me awake if anything… I don’t know if those sorts of things help necessarily if you’re agitated about dying, I’m not sure if taking such medication is helpful. (Maria, patient, 45-54)

Experience of sedation monitoring, the minor sub-theme arose with only four participants, who mainly mentioned clinical observation and responses to verbal stimulation as monitoring methods:

Every time they *[clinical staff]* came in the room they wrote things down. They’d come in, have a look, and give her a bit more, if it was needed. (Pauline, bereaved relative, 45-54)

In my wife’s case, they came in and they would ask her “Can you hear me?” and that’s how it went. They’d see if she responded and her general behaviour, and they kept her calm to the very time that she died. (Charles, bereaved relative, 75+)

### Theme 2: attitudes towards BIS

Discussions chiefly focused on exploring participants’ perceptions of BIS. This theme therefore had most sub-themes and categories. We identified three sub-themes: positive perceptions, conditional acceptance, and reservations (Table 3).

**Table 3:** Attitudes towards BIS: sub-themes and categories

Attitudes towards BIS
**Positive perceptions**
• Potential benefits
• Would be acceptable to use for themselves or their family member
• Appearance of sensor strip/monitoring screen acceptable
• Any helpful intervention is acceptable
• Potential for use alongside other monitoring methods
**Conditional acceptance**
• As long as patient and/or family involved in decision to use BIS
• Depending on patients’ individual characteristics/symptom severity
• As long as it is clinically useful
• As long as it is used only in addition to usual care
**Reservations**
• Medicalisation of care and/or death
• Appearance of sensor strip/monitoring screen
• Unreliable readings
• Movement restriction
• Skin irritation
• Misinterpretation of readings
• Potentially invasive
• Providing information which could be distressing for family members
• Used for continuous sedation until death
• Inappropriate for agitated patients

#### Positive perceptions

Most of our participants considered that, if BIS monitoring were offered to them or a family member in a palliative care setting, it would be acceptable. They said that it might be useful for clinical teams, commenting that BIS could enable systematic monitoring of patients’ consciousness levels, and aid adjusting medication to suit each patient’s individual needs:

Primarily it’s actually information for the [*clinical*] teams themselves. I mean, to me, it would be interesting because we are having to do so much by guesswork at the moment. I have a feeling that Mum thankfully has a lot less pain now than she might have had a few weeks ago. So, I’ve asked the question of “Can we try slightly less medication?”. It seems to be something that the monitoring system would be able to provide perhaps… a more accurate way of understanding what level of sedation she’s at and whether that’s appropriate for what her needs are at the time. (Liz, relative of current patient, 35-44)

Most participants commented that additional information from BIS might assist clinicians to ensure patient comfort, and possibly improve it. They considered this one of the main potential benefits for incorporating BIS into clinical practice, especially for people who were no longer able to communicate and might be experiencing pain or suffering at the ends of their lives:

I hope they start using it in practice and you know, save a lot of people’s suffering [*at the*] end of their life, because [*at the*] end of our life [*it*] doesn’t matter who I know, who comes to visit me or anything… [*We are*] all probably ourselves, we won’t be able to say how much the pain [*is*]. Once we can’t talk… this machine will communicate between we [*sic*] and the professional person. It seems to me there’s a technology there helping professional people how to comfort us. (Sheba, patient, 65-74)

Participants felt that BIS was non-invasive, unlike some other medical interventions. This was important for perceiving BIS as acceptable in palliative care. Some participants explicitly described that the intrusion caused by the monitor would be minimal, comparing it to other wearable technological devices:

It’s not invasive so I have no problems with it whatsoever. If it was invasive like sticking a needle in your arm or a bit like a cannula, I might get a different answer but it’s not invasive. It’s just stuck to your skin, it’s almost like wearing your, what they call now, smart watch. (Archie, patient, 65-74)

Most participants considered the appearance of the BIS monitor and sensor strip acceptable. They described them as small or discreet, and therefore unlikely to be noticeable to patients or visitors:

The monitor seems quite discreet really and the strip is pretty small. Once it’s been put in place, I don’t imagine they *[patients]* would be aware of it, you would probably not notice it. (Ellie, bereaved relative, 65-74)

Patient relative participants particularly emphasised a potential benefit which they perceived for BIS: its potential to enable continuous monitoring of patients’ level of consciousness, which is not possible with physical observations alone. These participants commented that, if BIS were part of patient care, they would feel, or would have felt, relieved and reassured that their family member was comfortable, and having their needs met:

In my experience, I think the nurses, if you’re in a hospital, they’re so busy you know… If people can’t be physically monitored 24 hours a day, I think it might be reassuring to know “Oh well, they’ve got the monitor and they are being checked on and they are comfortable”. (David, bereaved relative, 45-54)

In broader comments beyond those specifically responding to questions from the topic guide, our participants expressed their general disposition towards medical/technological interventions in palliative care. In these wider reflections, many stated that “anything” that could help patients to become more comfortable at the end of life would be acceptable:

I mean, from a personal point of view, anything that a clinician can use to help me at end of life to be comfortable… Going from the state of health I’m in now to the state I’ll be in at the end, it’s this journey, if you like… If it’s an unpleasant journey, anything that helps to make it a pleasant one, is to be desired. (Archie, patient, 65-74)

I think my mum would have said “Yes, do anything you need to do”. She would say, “Just hook me up to anything”. I think she would have just welcomed anything that could have helped her. (Pauline, bereaved relative, 45-54)

#### Conditional acceptance

Some patients and relatives felt that using BIS in palliative care would be acceptable as long as certain conditions were met. These mostly related to the clinical usefulness of the technology, its role in complementing, rather than replacing, usual care and practice, and patients’ consent to its use.

In response to the researchers’ introductory remarks on the lack of research on BIS in palliative care, some participants commented that, if further evidence indicated that BIS monitoring was beneficial, they would have no objections to its use, if that was in addition to usual practice, rather than replacing clinical observation and decision-making:

I mean, as long as there’s a medical advantage, I just can’t see any reason to not use it… to not use it as an aid. (David, bereaved relative, 45-54)

If it’s a tool to aid the care but it’s not the only thing then I think that’s fine… If it’s used as a guide, as a tool, as an extra, then fine. Βut there’s nothing that beats, you know, somebody walking into a room and going ‘Oh goodness, I think we need to do something here’. (Liz, relative of current patient, 35-44)

Participants also felt that patients should be informed of the option of receiving BIS monitoring and having its potential benefits explained by clinical teams, so that they could give their consent to its use, ideally before entering the final stage of life. Alternatively, if patients were unable to consent, family members should be consulted on whether BIS should be included in their care:

I think with all of this, it’s all good… but it’s important to let the patient choose as much as possible, like before they get into a state of being unconscious, to make sure you’ve had this conversation and that they’re aware of these things. Αnd you can ask the patient “If it comes to the point where you are not conscious, would you like to be monitored?”. It should really be up to the patient, and if the patient can’t decide, then possibly the family. (Maria, patient, 45-54)

#### Reservations

Some patients and relatives indicated that they had reservations regarding the use of BIS in palliative care. These mainly pertained to the employment of medical/technological interventions at the end of life, and the appearance of the BIS sensor.

A few participants expressed the reservation that using BIS with dying patients would be opposed to their understanding of hospice care, by increasing medicalisation of dying, and thereby decreasing the possibility of a more “peaceful” death:

Obviously, I’m not against technology. I just would like it to be as calm and peaceful as possible. And I think that, on the whole, that’s what hospices are really good at, they’re good at pain management, they’re good at making you comfortable, they’re good at letting you alone, and so I wouldn’t want anything more than the kind of basic minimum. (Julie, bereaved relative, 65-74)

I think there’s always this image in mind to try and make dying as comfortable as possible and somehow that’s a bit separate… a slight step away from that, the vision of having a nice, peaceful end of life.

(Rob, patient, 65-74)

A few participants, contrasting with the more positive views of others, felt that applying the sensor strip to the patient’s forehead made BIS more overt and noticeable than other monitoring methods. One commented that the sensor did not “look very good”, and could be made to look “cool” or “more sophisticated”:

I’ve worked with these brain things, they never look very good, you’d hope that it gets something a little bit, you know, cool… so it looks cool… doesn’t look as if you’re putting an electrode on someone’s head. There’s always a bit of a horror about that kind of thing. So, maybe something slightly more sophisticated might look better. (Rob, patient, 65-74)

Other participants said that the sensor could be covered with a cap, or a hat or “beanie”, or made to look “prettier” with decoration:

Well, stick it *[sensor strip]* inside a New York Yankees baseball cap or something… or a beanie, or whatever… There’s a lot of beanies in cancer. (Matthew, bereaved relative, 45-54)

I wonder if there’s something you could sort of make that it’s not so obvious? Maybe like a cap or something so that it doesn’t look like this… Maybe you could make it look prettier, put some flowers on them [*sensors*], but it’s very clever. (Pauline, bereaved relative, 45-54)

### Theme 3: Acceptable durations and settings for BIS use

#### Duration

Patients and relatives mostly felt that the duration of BIS monitoring should be determined by the stability and severity of patients’ symptoms. When asked about acceptable duration of monitoring, they responded that BIS should be used for “as long as necessary”, provided that people were not distressed by having the monitoring strip attached to their foreheads. When asked about when monitoring should stop, opinions varied (see Table 4). Some participants preferred the idea of continuous monitoring until the very end of life:

As soon as the person is starting to receive sedation, right up until possibly the day they die because you’ve got then a whole picture in front of you of what’s actually happening… I would say, that would give you the most valuable feedback. (Bobbie, bereaved relative, 45-54)

**Table 4:** Acceptable durations and settings for BIS use

When and where?
**Duration**
● As long as necessary
● As long as not causing distress to patient
● Patient decision
● Clinical decision
● Intermittent use – Until appropriate doses of medication established
● Continuous use – Until the end of life
**Location**
● Acceptable in all settings
● Particularly useful for home care patients

Other participants thought it would be better to use the device intermittently, suggesting attaching BIS when patients’ conditions changed, and removing it once patients were comfortable:

I can’t see it being necessary to sort of have it on permanently. If you’re on it for a day for instance, while they’re *[clinical staff]* determining what your dosage needs to be… Once they’ve made a decision as to what dosages you need to keep you comfortable, you wouldn’t need that on anymore unless something changes and then you can be back on it for them to decide again the dosages. (Archie, patient, 65-74)

#### Settings

When asked about the settings or locations where BIS monitoring would be acceptable, most participants said that this would be anywhere where people receive palliative care. Some added that BIS would be particularly useful for home care patients, who are mainly looked after by informal carers without clinical training. Participants commented that in patients’ homes BIS might help in guiding the administration of medication, if home carers were trained in using the technology and interpreting BIS readings, and clinical support was available:

I think it should be used everywhere. If it works and it helps people, then... I mean, it’s not, it’s not a big bit of kit, is it? Plug it in and strap it on. It’s not a large device, so yeah, use it at home, use it here [hospice], use it in the hospitals, use it where you can. (David, bereaved relative, 45-54)

At home, I think to help district nurses or to anybody that’s caring for somebody that’s able to administer medication, I think it would be invaluable, yes. To know, especially in those like last few weeks, am I giving too much, too little, you know, what’s a good rate? But without any of the monitoring on… nobody’s gonna know so I’m all for it. (Bobbie, bereaved relative, 45-54)

## Discussion

This study aimed to explore the perceptions of UK palliative care patients, relatives of current patients, and bereaved relatives about the potential use of BIS technology in palliative care. Our study participants generally considered that it would be acceptable in principle to monitor palliative care patients’ levels of consciousness using BIS technology. This is largely consistent with the limited evidence on the acceptability of BIS from previous studies in other countries [36, 38–40], and also with studies of other health care technologies, which have found that patients and carers tend to be open to using novel technologies [23, 24]. We found no major differences in perspectives between patients, current carers, or bereaved carers, although they varied in their prior knowledge and/or experiences of using sedative medication, and their overall attitudes towards the use of medical/technological interventions in palliative care.

Some participants expressed some reservations about BIS, but none were wholly negative towards it. Most of our participants remarked that BIS might be acceptable in end-of-life care, as long as patients and/or relatives consented to its use, and if it was used in addition to usual care practices, complementing, rather than substituting, clinical observation and care. A study of palliative care patients’ and carers’ perceptions of telehealth applications found similar responses from participants, who commented that such initiatives should be offered as a supplement to existing practice [25].

Some of our participants voiced concerns that BIS monitoring could medicalise palliative and end-of-life care, and a few of those considered that the presence of the sensor on the patient’s forehead made BIS monitoring more noticeable than other palliative care interventions. Some offered suggestions such as using accessories or headwear to cover the sensor. However, most of our participants considered that BIS was less invasive than other medical technologies, and compared it to wearable personal technologies. These participants commented that the sensor was discreet and therefore acceptable for use with palliative care patients, and that, because of this, they considered that it was unlikely that BIS monitoring would negatively affect patients’ care experiences. This resonates with a recent study investigating the use of monitoring devices for sedated palliative care patients, in which no family members raised issues with how those devices looked [41].

Most of our participants considered that the potential for continuous, systematic monitoring of consciousness levels was one of the main potential benefits of BIS. They observed that, if this type of monitoring captured real-time changes in patients’ conditions, it might assist in timely, individualised administration of sedatives, which they thought might in turn help improve symptom control and patient comfort. Some of these participants also felt, similarly to findings from other research studies on remote technologies [24], that monitoring patients’ consciousness levels at home, when health professionals were unable to physically observe them, was potentially reassuring for home carers, and would assist with their anxieties and uncertainties. It is important to note, however, that the reassurance provided could be misleading, and potentially result in risky assumptions that the patient was not experiencing any problems.

Our findings, alongside other research on relatives’ perceptions about the use of medical technologies in end-of-life care [20], suggest that, as with other technologies, if BIS were to be introduced into clinical practice it should be accompanied with careful information and guidance for patients and relatives about its technical aspects and its potential role as an adjunct to usual care.

### Strengths and limitations

A strength of our study is that it included current patients and current and bereaved relatives, with varied backgrounds and service use experiences, enabling perceptions from all these different groups to be explored. These findings were sufficient for our initial purposes, enabling us to determine that it would be acceptable to proceed to explore the use of BIS with patients in UK hospice practice. Our findings also confirm similar findings from the few previous studies which have investigated the use of BIS with patients receiving end-of-life care in clinical settings in countries other than the UK [36, 38–40]. These studies also found that BIS was acceptable to patients and their families as an adjunctive tool for monitoring consciousness levels, and that participants expressed feeling supported and comforted that BIS was part of their/their family member’s end of life care [36, 38–40].

Since our study included a relatively small number of participants recruited from one UK hospice, our findings may not be directly generalisable or transferable to other settings or countries. In addition, lack of prior research in this field meant that we did not know which characteristics of participants might be relevant for our study. We therefore could not identify specific characteristics by which to conduct any purposive sampling, although we note that purposive sampling can be challenging when conducting research with patients receiving end-of-life care, whose status can change suddenly and unexpectedly. Finally, our study participants were self-selected from among those patients and relatives who met the inclusion criteria. It is possible that these people had more positive attitudes towards the use of medical technologies in palliative care in general, and might therefore be more likely to find BIS monitoring more acceptable.

## Conclusions

This is the first study in the UK to systematically explore the views of palliative care patients and their relatives regarding the acceptability of using medical technologies based on electrophysiological data, such as BIS, in advance of any introduction of this technology in practice. Our study participants considered BIS technology to be a potentially beneficial, non-intrusive means of assisting clinical assessment and decision-making at the end of life. Some participants had some relatively minor reservations, but in general they thought that trialling BIS technology would be acceptable, and that its eventual use in practice might be acceptable, particularly if patients and/or family members were involved in decisions about its use, and if it were to supplement, rather than replace, usual care. Since this study with current patients and current and bereaved relatives did not identify any serious objections to trialling this technology with patients, our Study Advisory Group agreed that it was acceptable to proceed to the next stage of the study and to trial BIS in practice. This enabled us to begin exploring the under-researched feasibility and clinical utility of BIS in palliative care in the UK. Future papers will report on this subsequent research.

## Supplementary Information

**Additional file 1.** Interview/focus group topic guide (minor adaptations made for each participant group as appropriate.

## Data Availability

The datasets generated and/or analysed during the current study are not publicly available due to small numbers and data protection issues but are available from the corresponding author on reasonable request.
