# Supplementary material for: Do palliative care patients and relatives think it would be acceptable to use Bispectral index (BIS) technology to monitor palliative care patients’ levels of consciousness? A qualitative exploration with interviews and focus groups for the I-CAN-CARE research programme
Source: BMC Palliat Care. 2022 May 24;21:86. doi: 10.1186/s12904-022-00949-w (PMC9131519; doi:10.1186/s12904-022-00949-w)
Supplement: Supplementary file 1 — Additional file 1. Interview/focus group topic guide (minor adaptations made for each participant group as appropriate. [file 12904_2022_949_MOESM1_ESM.docx]

**Additional file 1.** Interview/focus group topic guide (minor adaptations made for each participant group as appropriate)

| 1. **Preamble** 2. **Explanation of sedation** 3. **Presentation of BIS**   **Questions (plus prompts, if needed)**   1. Do you have any prior knowledge or experience of the use of sedative medicines?    1. Have you ever discussed the use of sedatives with anyone before today?    2. Do you have any prior knowledge or experience of how levels of sedation are monitored? 2. Do you have any thoughts about how the technology we have shown you might be used in palliative care?    1. What might be the benefits/advantages of using something like this in palliative care?    2. What might be the disadvantages?    3. If you personally needed to be sedated, how would you feel about your level of sedation being monitored using a device like the one we have shown you?    4. How would you feel if a relative needed to be sedated and their level of sedation was monitored using a device like this one?    5. Do you think this kind of device should be used in the same ways, or in different ways, for people in hospital, in a hospice, or at home? 3. If a person receiving palliative care is given sedative medicine, would it be acceptable to use this kind of device to monitor that person for the whole time they are sedated?    1. How long do you feel it would be ok to monitor you or a relative using a technical device like this?       - *For minutes; for hours; for days?*       - *As long as necessary?*       - *For as short a time as possible/just long enough to assess how deeply sedated the person is?* 4. Any other comments?   **8. Closing and thanks** |
| --- |
